# Management of Patients With Hematologic Malignancies During the COVID-19 Pandemic: Practical Considerations and Lessons to Be Learned

**DOI:** 10.3389/fonc.2020.01439

**Published:** 2020-08-14

**Authors:** Alessandro Isidori, Laurence de Leval, Usama Gergis, Pellegrino Musto, Pierluigi Porcu

**Affiliations:** ^1^Hematology and Stem Cell Transplant Center, AORMN Hospital, Pesaro, Italy; ^2^Institute of Pathology, Lausanne University Hospital and University of Lausanne, Lausanne, Switzerland; ^3^Division of Hematologic Malignancies and Hematopoietic Stem Cell Transplantation, Department of Medical Oncology, Sidney Kimmel Cancer Center, Thomas Jefferson University, Philadelphia, PA, United States; ^4^Unit of Hematology and Stem Cell Transplantation, Department of Emergency and Organ Transplantation, “Aldo Moro” University School of Medicine, AOU Consorziale Policlinico, Bari, Italy

**Keywords:** SARS-CoV-2, hematologic malignancies, COVID-19, lymphoma, myeloma, leukemia, CAR T-cell, telehealth

## Abstract

The COVID-19 pandemic has created unprecedented hurdles to the delivery of care to patients with cancer. Patients with hematologic malignancies appear to have a greater risk of SARS-CoV-2 infection and severe disease due to myelosuppression and lymphopenia. The first challenge, therefore, is how to continue to deliver effective, curative therapy to vulnerable patients and at the same time avoid exposing them, and their health care teams (HCT), to SARS-CoV-2. An additional challenge is the timely completion of the diagnostic and staging studies required to formulate appropriate treatment plans. Deferred procedures and avoidance of multiple trips to the surgical, diagnostic, and laboratory suites require same day consolidation of all procedures. With laboratory medicine absorbed by the need to deploy large scale COVID-testing, the availability of routine molecular tests is affected. Finally, we are increasingly faced with the challenge of making complex treatment decisions in SARS-CoV-2 positive patients with aggressive but potentially curable blood cancers. When to treat, how to treat, when to wait, how long to wait, how to predict and manage toxicities, and how to avoid compromising cure rates remains unknown. We present an outline of the scientific, medical, and operational challenges posed by the COVID-19 pandemic at selected American and European institutions and offer our current view of the key elements of a response. While the peak of the pandemic may be past us, in the absence of a vaccine risks remain, and our alertness and response to future challenges need to be refined and consolidated.

## Introduction

In December 2019, an outbreak of atypical pneumonia, with severe acute respiratory syndrome (SARS), was identified in Wuhan, China (1, 2). The rapid human-to-human transmission of the new zoonotic coronavirus (SARS-CoV-2), that is responsible for what is now known as coronavirus disease 2019 (COVID-19) led to a global outbreak, which on March 11, 2020 was declared a pandemic by the World Health Organization (WHO). The COVID-19 pandemic is an unparalleled challenge for the medical community and has created a multitude of medical, logistical, financial, and public health challenges in the delivery of optimal care for cancer patients, and in particular for patients with hematologic malignancies.

Patients with leukemia, lymphoma, and myeloma frequently visit the hospital or the outpatient clinic for treatment, transfusion support, antibiotic therapy, management of complications, and disease surveillance. In addition, these patients often require prolonged hospitalizations, myelosuppressive chemotherapy, and the use of high-risk procedures such as autologous or allogeneic Stem Cell Transplantation (SCT). Therefore, these patients are generally immunocompromised due to the underlying malignancy and/or anticancer therapy and are at higher risk of developing infections (3).

Initial reports from China suggested that patients with cancer had an estimated two-fold increased risk of contracting SARS-CoV-2 than the general population and, if infected, also had a higher risk of severe events (ICU admission, invasive ventilation, or death) compared to patients without cancer (4). These risks are likely to be greater in patients with hematologic malignancies, leading to an urgent need to address the impact of the COVID-19 pandemic on the care of these patients. Challenges include equitable resource allocation, delivery of clinical care without undue risk of exposure to patients and health care workers, and the consent process during a time of restricted travel and isolation.

Standard management of cancer patients has been impacted first by efforts to limit exposures and new cases during the acceleration phase (treatment while under a regime of isolation), then by limited access to healthcare resources during the “surge” period (deferment management), and finally by an after-surge of new diagnoses, recurrences, and disease complications that require initiation of treatment (post-surge planning for deferred patients).

By searching PubMed for *COVID-19* and *leukemia, myeloma*, or *lymphoma* we found approximately 40 papers that matched. Data are limited, but the outcomes of patients with cancer who are COVID-19-positive at this time appear to be worse than for COVID-negative patients (4), including patients with hematologic malignancies.

The largest experience on COVID-19 in patients with hematologic malignancies so far was reported by He et al. (5). One-hundred and twenty-eight patients with hematologic malignancies, hospitalized at two centers in Wuhan, China were evaluated in a cohort study; 13 (10%) developed COVID-19. Moreover, 226 health care providers were studied, 16 of which developed COVID-19, and 11 were hospitalized. No significant differences in baseline characteristics were observed between the patients with hematologic malignancies who developed COVID-19 and those who did not. However, COVID-19 appeared to be more severe, and more deaths were reported, in the patients with hematologic malignancies compared to the cohort of healthcare providers who developed COVID-19.

Several papers have appeared, by individuals, groups of specialists or under the auspices of scientific societies, to offer an initial description of the clinical presentation of COVID-19 in patients with hematologic malignancies and to advocate for general recommendations of good clinical practice in this pandemic period, for both adult and pediatric patients (6–21). One interesting case report involved a SARS-CoV-2 infection in a 39-year-old patient with chronic lymphocytic leukemia (CLL) where the clinical and biochemical manifestations of COVID-19 were partly masked by the coexisting CLL (6). One letter by the University Hospital of Dublin reported on the positive outcome of a young woman with refractory Hodgkin lymphoma (HL) on pembrolizumab who wasinfected with SARS-CoV-2, required intensive care service without intubation, and was safely discharged from the hospital after 16 days (7). A French group (8) reviewed the data of 25 consecutive adult patients admitted to a Hematology Department in Paris with confirmed COVID-19 infection and at least 10 days of follow-up. Twenty patients had a lymphoid malignancy including 10 patients with multiple myeloma (MM); there was relative overrepresentation of MM patients in that cohort compared to the usual activity, suggesting that MM patients might have a higher risk of developing SARS-CoV-2 infection. Of the 18 patients with a follow-up, 52% developed acute respiratory distress syndrome (ARDS), and 6 required mechanical ventilation; 9 of the patients with ARDS died. Of note, many patients were older (>65 years), with one or more comorbidity, which could have increased the severity of COVID manifestations.

Specific guidelines have been proposed for chimeric antigen receptor T-cell (CART) therapy (9), acute myeloid leukemia and myelodysplastic syndromes (10–14), infections (15), chronic myeloid leukemia (16), chronic lymphocytic leukemia (17), and use of BTK inhibitors (18), which may have the potential benefit of blunting the hyperinflammatory response to SARS-CoV-2, but also potentially increasing the risk of secondary infections or impaired humoral immunity (18).

For multiple myeloma patients, different papers proposed recommendations specifically adapted to their management during the COVID-19 pandemic, stemming from expert panels or individual expert opinions (22–24).

Notwithstanding this plethora of papers, a practical, comprehensive guide for clinicians involved in the path to cure of patients with hematological malignancies is still lacking and thus is urgently needed, especially if the number of COVID-19 positive cases increases worldwide and the lockdown of many countries continues.

In this article, we attempt to provide some practical suggestions on the management of patients with hematologic malignancies during the COVID-19 pandemic. The manuscript briefly covers practical issues such as delays in diagnosis, deferral of chemotherapy, utilization of SCT, modifications of maintenance treatments, access to and utilization of supportive measures, role and safety of targeted therapies, as well as ways to mitigate these changes. Moreover, we will try to answer some burning questions, such as: (1) Who and when should be tested? (2) Is multiple testing needed? (3) Which therapies should be considered? (4) What about older patients? (5) Should SCT (autologous and allogeneic) be delayed?

## Diagnosis and Testing

The diagnostic facilities of clinical medicine and pathology laboratories have also been directly or indirectly affected by the pandemic. The urgent need for developing reliable tests for SARS-CoV-2 infection and performing these analyses quickly and reliably on a large scale have required the reorganization of the activity of technicians and scientists to increase and optimize the resources dedicated to viral testing. At the same time, pathology departments have generally been facing a decrease in their total caseloads in surgical pathology and cytology, because elective surgeries were canceled in most hospitals and clinics, and ambulatory consultations were reduced to a minimum. More specifically however, in line with the selection of patients eligible for surgery, there was an enrichment in complex surgical cases or critical diagnoses. In addition, several pathology departments in academic centers and large institutions have implemented an autopsy program for COVID-19 patients, imposing the application of strict biosafety precautions as recommended by the CDC and other national societies, and requiring the allocation of internal resources to that activity.

The laboratory diagnosis of hematologic malignancies typically relies on multiple parameters starting with morphology provided by the cytological examination of smears or histopathologic assessment of tissues, with the addition of immunophenotypical, molecular and genetic studies based on the type of disease and its complexity. It requires the intervention of certified pathologists or cytopathologists, clinical scientists, and technicians. In different areas of the world where the epidemics has been variably developing, variable rates of infection among the medical and paramedical staff have been reported, with direct consequences on the medical and diagnostic operations. Moreover, in several institutions a rotating service among the personnel has been implemented to allow social distancing of the staff in the working places, and to preserve part of the staff in case of an infection outburst.

Besides intra-laboratory factors, the main factor impacting the diagnosis of hematologic malignancies is the acquisition of diagnostic samples. In case of clinics or medical practices using the services of outside laboratories, shipment of the samples may be delayed. Probably the main challenge, for several reasons detailed in the other sections, is that delay in spontaneous presentation or referral of symptomatic patients, and these may further face limited medical availability implying additional delays. While the feasibility of blood drawing and blood testing is in principle least compromised, bone marrow aspiration, and biopsy which require qualified operators and assistants, is possibly avoided in situations where it is considered not absolutely necessary at first sight, potentially eliminating the availability of important information. One major problem is the performance of diagnostic biopsies of lymph nodes or other masses suspicious for lymphoma. Operating rooms have been largely shut down, with the limited available access restricted to patients requiring urgent, life-saving interventions, according to the recommendations proposed by national societies or institutional policies. In that setting, deferral of the biopsy or alternatively fine needle aspiration (FNA) biopsies, can be proposed. At this moment, we lack information to draw conclusions on the potentially deleterious consequences this situation has engendered. It will be of interest to analyze both the pandemic period and the post-surge period for any differences in the incidence of new diagnoses of hematological malignancies, both globally and with respect to indolent and aggressive types, and to look into whether the deferral of diagnosis correlates with more advanced stages of disease at initial presentation.

One of the many consequences of the COVID-19 crisis has been on clinical trials with restrictions in continuing to enroll new patients and conduct the clinical trials, including completion of trial visits and trial assessments. This is due to multiple factors both on the side of the patients and of the healthcare system: self-isolation and restrictions of visits to healthcare facilities required for vulnerable individuals including some trial patients, clinics allowing only essential or critical visits or refusing to take part in trials, recruited patients dropping out of trials, the background of increased pressure on the health service changing trial staff availability. There may be a need for critical laboratory tests, imaging or other diagnostic tests to be performed for trial participant safety or per synopsis of the trial, which cannot be completed as anticipated. In cases the trial participant cannot reach the site to have these performed, or the biological samples cannot be shipped to the central reference laboratory, the tests may be performed in another local facility. Competent health authorities (European Medicine Agency, Swissmedics, and the Food and Drug Administration) have issued guidelines for handling the effects of COVID-19 on clinical trials to maintain the integrity of the trials, to ensure the rights, safety and well-being of trial participants and the safety of clinical trial staff during this global public health crisis.

The emergence of the pandemic has highlighted the need to promote innovative uses of technology to compensate the reduction or prohibition of in-person meetings and to limit the exposure risks to health care providers, patients, and the community. Many cancer centers were forced to cancel tumor board meetings and went to virtual meetings using web-based or institutional platforms for communications. In several pathology departments equipped with slide scanning instruments, pathologists have been able to review slides remotely and to provide diagnoses from their homes. While digital technology is increasingly used for the communication of histopathological slides, its application to cytological smear preparations has not yet been widely reported. It will be of interest to analyze in the near future what we have learned from the “remote” experience during this period, and which positive points can be retained for future practice or in case of future healthcare crises.

## Management of Patients With Acute Leukemia

The treatment of acute leukemia differs significantly between the United States, in which many novel drugs are FDA approved or available in clinical trials, and the rest of the world. Moreover, while in the US intensive induction with daunorubicin and cytarabine in selected younger patients can administered in an outpatient fashion, in Europe patients are still hospitalized until full hematological recovery. Both the American Society of Hematology (ASH) and the European Hematology Association (EHA) have released short preliminary guidelines to assist with the management of some practical issues in AML patients during COVID-19 pandemic (25, 26). In this chapter, we will try to briefly summarize the most important components of diagnosis and treatment, during induction, consolidation, and maintenance.

### Diagnosis

1. All patients with newly diagnosed acute myeloid leukemia (AML) or acute lymphoblastic leukemia (ALL) should have a COVID-19 test before admission. As serological tests still do not have high specificity and sensitivity, the preferred test is RT-PCR on oropharyngeal/nasal swabs. It is mandatory to wait for the results before admitting patients in rooms with positive pressure, in order to avoid the diffusion of the virus among patients in case of a patient with positive swab.
2. Approximately 50% of the patients with AML/ALL have fever at diagnosis. Patients with fevers and a blood count and a peripheral blood smear highly suspicious for acute leukemia must have a COVID-19 test, and possibly be isolated in a single room with negative pressure while waiting for the results. COVID-19 negative patients should move to the hematology division as soon as the results are available, in order to perform the diagnostic procedures and start chemotherapy.
3. Bone marrow aspiration and biopsy in all symptomatic patients with a pending COVID-19 swab test must be performed wearing personal protective equipment (PPE) with contact and droplet precautions, including eye protection.

### Induction and Consolidation

1. Induction and consolidation therapy in patients with acute leukemia are associated with a high risk of infectious complications, due to the prolonged neutropenia that follows chemotherapy. Accordingly, AML and ALL patients should have a COVID-19 test before each treatment cycle, even in the absence of clinical symptoms.
2. In some centers, chemotherapy may have to be delayed or deferred if the patient tests positive for SARS-CoV-2 or due to a shortage of isolation beds and/or blood products.
3. A delay of induction chemotherapy by 7–14 days in AML patients waiting for results of COVID-19 testing is acceptable during the pandemic, based on recent data presented by the German group (27).
4. AML patients suitable for intensive induction and with a negative COVID-19 test should be hospitalized in single room with reverse isolation and treated with standard induction. Visitors should not be allowed during the whole hospitalization, in order to minimize the risk of COVID-19 infection during the hospital stay.
5. Treatment for AML patients not eligible for intensive therapy should start only if COVID-19 negative. In patients with rapid progressive disease, hydroxyurea 2–6 g daily can be used to reduce the tumor burden while waiting for swab results.
6. The combination of venetoclax and hypomethylating agents (HMAs) at present is approved by the FDA but not by the EMA. This treatment, approved for the elderly, is usually administered in an outpatient setting. Nonetheless, this combination seems to induce more prolonged bone marrow aplasia than HMAs alone. Accordingly, it should be used with caution during the COVID-19 pandemic.
7. The association of arsenic trioxide (ATO) and all-trans retinoic acid (ATRA) in patients with acute promyelocytic leukemia (APL) may result in severe respiratory distress during induction. Accordingly, idarubicin and ATRA are preferred for induction during COVID-19 pandemic, followed by ATO + ATRA consolidation, in order to reduce the relapse rate in patients with low-intermediate risk profile.
8. Patients with ALL receiving inpatient treatment after testing negative for COVID-19 can be approached as patients with AML. While lymphopenia is considered predictive of worst outcome in COVID-19 patients without hematological disease, this is yet to be demonstrated in ALL patients.
9. The addition of tyrosine kinase inhibitors (TKI) to standard chemotherapy in Philadelphia positive ALL does not result in increased infectious toxicity, and thus should be continued in COVID-19 negative patients.
10. The use of dasatinib as maintenance therapy post-allogeneic SCT increases the risk of CMV reactivation and pleural effusion (28). Accordingly, dasatinib should be used with caution during the COVID-19 pandemic (9).
11. COVID-19 positive patients with acute leukemia at any phase of the disease (diagnosis, complete remission, relapse) should be managed in dedicated COVID-19 unit if symptomatic, or at home if not symptomatic. Even if conclusive evidence is still lacking, empiric therapy with hydroxychloroquine, azitromicin, and enoxaparin (if platelet count over 30 × 10^9^/l) is current Italian clinical practice.
12. The equitable utilization of scarce ICU resources for patients with acute leukemia and severe COVID-19 pneumonia requiring life support during the pandemic, remains a very challenging ethical dilemma, which health systems from different countries have approached in different ways. Patients who are receiving induction with curative intent and patients who develop COVID-19 while in complete remission should be offered ICU care. For patients with no curative options, relapsed disease, and significant comorbidities, the decision has to be individualized.

### Relapse

1. Patients with acute leukemia in first or subsequent relapse should start the treatment only if COVID-19 negative.
2. For patients with relapsed disease, the treatment guidelines of each center should be followed. If possible, enrollment in a clinical trial targeting genetic abnormalities is strongly encouraged.
3. At relapse, molecularly targeted therapy for AML or blinatumomab/inotuzomab based therapies for ALL patients should be preferred.
4. Stem cell transplantation after reinduction is still considered the best treatment option in COVID-19 negative patients with acute leukemia who did not receive it as a part of their front-line treatment.

### Maintenance

1. The vast majority of protocols for ALL include 2 years of maintenance therapy after induction/consolidation. As of March 19th, 2020, GRAALL-14 investigators decided to skip vincristine and prednisolone during maintenance, whilst continuing 6-mercaptopurine and methotrexate.
2. Maintenance with HMAs in patients with AML not suitable for intensive chemotherapy achieving either a complete or partial response or a stable disease should be continued up to progression, given the extremely poor outcome for patients discontinuing HMAs.

## Management of Patients With Multiple Myeloma

Multiple myeloma (MM) patients are generally old, frequently frail, and display clinical features, including a poor functional status, that lead to a high risk of severe COVID-19 infection and death. MM patients have high rates of co-morbidities, particularly diabetes, hypertension, chronic obstructive lung disease, and cardiac diseases, sometimes second malignancies. They also differ in their urgency of treatment and in intensity of therapy. Furthermore, the immune system in patients with MM is compromised at diagnosis by several factors, which include paucity of functional immunoglobulins and decreased CD4+ T-cell count. Importantly, low rates of seroconversion have also been documented in influenza and pneumococcal vaccination in MM patients. Moreover, treatments for MM often cause even more immunosuppression: CD4 counts decrease after proteasome inhibitors (PIs) or monoclonal antibodies, whereas myelotoxicity, neutropenia, and decreased T-cell response, frequently follows therapy with immunomodulatory drugs (IMiDs).

Steroids, a backbone agent in most MM regimens, are well-known to exert immunosuppressive activity, particularly when combined with IMiDs and PIs. Lymphopenia may worsen after starting therapy with some monoclonal antibodies (i.e., elotuzumab). The slow and sometimes incomplete hematopoietic recovery and the prolonged immunosuppression observed in patients who receive autologous stem cell transplantation (ASCT) is another factor. All these findings make MM patients probably more susceptible to COVID-19 infection and support the assumption that they are at increased risk for poor outcomes if infected by SARS-CoV-2. While data confirming these assumptions are needed, it is important to decrease at the upmost the risk of SARS-CoV-2 exposure in MM patients.

There are still not sufficient data to produce evidence-based recommendations, so that only consensus statements have been so far published in this setting (22–24, 29–31). Therefore, we will provide here our personal suggestions, based on experience, on the management of MM patients during the ongoing COVID-19 outbreak.

### General Measures to Prevent COVID-19 Infection in MM Patients

Hospital admission and recurrent hospital visits increase the risk of COVID-19 infection, as asymptomatic individuals can transmit the virus. Therefore, efforts should be made to minimize patient exposure to COVID-19, by decreasing physical contact with the health care system, avoiding non-essential visits and minimizing the time spent by patients in infusion suites and other clinical areas. However, to avoid compromising patient's outcome by excessively postponing diagnosis or treatments, hematology departments should ensure full operative activity. Hematologists should actively elaborate protocols to control the spread of the infection through an adequate patient flow. All healthcare professionals should follow these procedures aiming to maintain “COVID-19 free” sectors, and reduce infection risk of other patients within the community of subjects followed at the same center.

As a general rule, we recommend COVID-19 testing (PCR) for all patients with newly diagnosed MM before starting any cycle of chemo or immune-therapy, before stem cell mobilization procedures and before ASCT. Treatment for MM, in fact, can worsen the symptoms of an active COVID-19 infection in these patients. Validated serological tests, could at some point also be useful for identifying patients who developed and recovered from COVID-19. Screening should be done for both outpatient and hospitalized patients, before initiating therapy. Healthcare professionals should also be quarantined and undergo COVID-19 testing (PCR) if a contact with a positive patient does occur. A positive PCR or a positive IgM or IgA test should be considered as “active COVID-19.”

Patient education is paramount. Social distancing, staying home (except for treatment), washing hands frequently with soap (for at least 20 s), covering coughs and sneezes with masks, and cleaning frequently touched surfaces represent pivotal and effective component of mitigating the risk of COVID-19 infection and are all strongly recommended.

Patients, staff and relatives should be triaged for symptoms and exposure to COVID-19 before entering the health care facility. Families, in particular, should be informed of the importance of reporting early respiratory symptoms, fever, or contact(s) with symptomatic people before having any interaction with the MM patient or close relatives. Medical visits, routine laboratory testing, catheter maintenance, and invasive procedures should be scheduled only if really necessary, and only for asymptomatic patients, in a COVID-free area. Implementation of telemedicine via audio or video technologies should be pursued to reduce patient exposure to SARS-CoV-2. Alternative and innovative methods of communication, such as bloodwork-monitoring at home/local laboratories or couriering of medications to patients' home may also be required to further minimize risks. Social support including tips for coping with stress should be also provided to the patient and their family members.

Regarding personal protective equipment (PPE), appropriate droplet-contact protections throughout the entire patient clinical assistance should be used, as SARS-CoV-2 can be transmitted, by asymptomatic carriers, before the beginning of clinical symptoms. Single use gloves are also recommended.

### Approach to Smoldering Multiple Myeloma

All patients with smoldering MM, according to current guidelines (32), should be monitored with no active intervention. In absence of symptoms or clear signs of laboratory/imaging evolution, tele-consultation should be the approach of choice. If possible, consider delaying with close follow-up if there is no imminent risk to the patients, such as diagnosis by SLiM criteria without any other end organ damage.

### Newly Diagnosed Patients Eligible for ASCT

High dose melphalan followed by ASCT is the treatment of choice for younger patients with newly diagnosed MM who are eligible for such a procedure (33). However, balancing the risks of COVID-19 infection and MM-related mortality is difficult and, therefore, in the current setting of the COVID-19 pandemic, deciding whether ASCT has to be performed, delayed, or excluded should be carefully evaluated on a case by case basis.

Although the necessity for intensive care after ASCT occurs only occasionally, it should be considered the risk of a possible shortage of ventilators and intensive care beds, putting MM patients who receive ASCT at increased risk, once they should require these interventions. COVID-19 outbreak could also negatively affect the availability of blood and platelet products, as the number of blood donors might decrease with the recommendation to self-isolate. Prolonged post-ASCT aplasia and, above all, the long time required for a complete immune reconstitution, should also be taken into account. Upfront ASCT for older adults (>70 years) with MM should be, at present time, omitted.

Several studies have evaluated upfront vs. delayed ASCT and, overall, they support the potential lack of a negative impact, at least on survival, of delaying ASCT (34–36). Thus, ASCT could be safely postponed for a pre-determined period of time for standard risk patients at least in very good partial response after induction therapy. In these cases, the prolongation of the treatment for up to six cycles may be a recommendable option. In standard-risk patients achieving a CR with or without negative minimal residual disease, initial therapy could be extended for up to nine cycles, delaying ASCT until the epidemic subsides or, alternatively, starting maintenance and preserving ASCT for the first relapse, as salvage therapy. In this scenario, however, it would be necessary to avoid treatments with stem cell poisons, in order to retain the ability to harvest hematopoietic stem cells afterwards. In patients with confirmed, recent COVID-19 infection, it is wise to delay stem cell collection; nonetheless, in some red zones, delaying stem cell collection should not be an option, but a necessity. Indeed, stem cells collection during front-line treatment may be not required for every patient. Conversely, patients with rapidly progressive disease, high cytogenetic risk (especially deletion of chromosome 17p), or those with plasma cell leukemia or extramedullary disease, should not postpone ASCT.

### Newly Diagnosed Patients Not Eligible for ASCT

Most elderly patients with MM are treated as outpatients, with frequent visits to the hospital (23). Risk stratification remains important, as well as the decision of whether the need for treatment is urgent or not. To optimize care of older patients with MM during this pandemic, decisions regarding dose-reductions, regimen modification/interruptions or continuation of therapy will need to be to be made, again, on a case by case basis. In this setting, while myeloma staging criteria (37) and geriatric assessment tools (38) have not been shown so far to assist with predicting for COVID-19 outcomes, they can be utilized for adapting the treatment of older adults with MM during this challenging time.

Patients with aggressive disease, defined by the presence of anemia, renal dysfunction, hypercalcemia, leukemic, or extramedullary presentation, or high-risk cytogenetics, need to start treatment as soon as they can, as they have a higher risk of dying for myeloma than COVID-19.

Induction treatment with regimens including only oral drugs should be pursued in frail patients (elderly and/or with comorbidities). These regimes require fewer visits (generally every 4 weeks), thus reducing the risk of SARS-CoV-2 exposure. Fit patients can be treated with standard therapies. We do not suggest any specific regimen, because available data regarding specifically COVID-19 and myeloma therapy are lacking. The choice of the treatment, which includes combinations containing bortezomib and/or lenalidomide, should be performed on the basis of previously established criteria, particularly the presence of renal failure, cytogenetics, thrombotic complications, extramedullary disease, or peripheral neuropathy. If any adverse event occurs, it should be considered dose reduction or dose delay, prolonging the interval between cycles. In particular, bortezomib should be administered weekly and using a subcutaneous route, and then every other week after 6–8 cycles, if a good and sustained response is obtained. Given the possible detrimental effect of steroids on patient outcome reported form previous coronavirus outbreaks, it should be considered to reduce the weekly dose of dexamethasone from 40 to 20 mg, to decrease the risk of infectious complications. In selected cases, in complete remission, dexamethasone should be omitted, while receiving continuous treatments. Finally, dexamethasone should be administered orally, in an outpatient setting.

Aiming to reduce potential exposure to COVID-19, we also recommend to decrease the frequency of blood work at the essential. In patient with adequate bone marrow reserve, blood work can be checked before starting the next cycle, rather than every week. Telemedicine should be the preferred way to monitor patients receiving treatments or in follow-up. Finally, pharmacists should be able to provide prescription doses for 2–3 months of treatment at a time, instead of the usual 1 month. Finally, home care, whenever possible, and switching from an intravenous or subcutaneous treatment to a fully oral treatment combination should be considered.

### Relapsed Patients

Most patients with MM relapse, but since not all relapses are the same, the main issue to consider is the time to initiate subsequent-line treatments. For clinical and more aggressive relapses, resulting in worsening end organ damage, subsequent treatment cannot be postponed. By contrast, active treatment for standard-risk patients, experiencing biochemical relapse, should be delayed. We do not recommend any specific regimen for relapsed patients, since no specific data is available on MM drugs and COVID-19, and thus only suggestions can be provided. Regarding daratumumab-based regimens, an infusion time of 90 min, after the third dose, in the absence of prior infusion reactions, may be used. Furthermore, after the achievement of at least a very good partial response, the subsequent schedule could be changed to a dose every 4 weeks instead of every 2 weeks. When the goals of therapy are achieved, and after 10–12 cycles, we suggest to consider switching to maintenance with lenalidomide. Carfilzomib should be administered once-weekly instead of twice-weekly in order to reduce the number of patients' admissions to health care units, depending on the combination regimen and dosing. Finally, fully oral treatment schedules should be preferred over intravenous or subcutaneous treatments, and switching from one kind of treatment (i.v. or s.c.) to another (oral) should be considered, on single case basis.

In relapsed patients supposed to receive ASCT as a part of the treatment plan, it should be carefully evaluated, on single case basis, to eventually delay transplant until the current pandemic resolves. For example, we recommend to proceed with ASCT without any delay in patients with aggressive relapse and suboptimal responses to salvage therapy). On the contrary, ASCT should be delayed in patients relapsing slowly and achieving deep responses with salvage therapy.

### Maintenance

Maintenance with oral drugs should be continued, without dosing modification and or interruption, in the absence major adverse events. If steroids are part of the regimen, a progressive tapering of the dose, until interruption, should be considered. Again, teleconsulting should be preferred for monitoring patients during COVID-19 pandemic. Blood testing should be performed by patients in the closest laboratory, and visits could be performed over the phone. Hospital visits should be performed every 3 months.

### Supportive Care

Anti-thrombotic prophylaxis (with a particular attention to the temporary use of heparin), as well as prophylaxis against varicella-zoster virus with acyclovir and against *pneumocystis jirovecii* with sulfa, remains unchanged as before the COVID-19 pandemic. Any additional anti-microbial prophylaxis is not recommended at the present time. Testing for other etiologies, such as influenza and other respiratory pathogens, may be required if symptoms appear. Vaccination, especially against influenza and pneumococcal species, is very important, as well as vaccination of family and contacts. Bisphosphonates such as zoledronic acid may be held for patients with stable disease and in the absence of significant bone related-disease. For other cases, we recommend using zoledronate every 3 months, rather than monthly, and the interruption in patients in complete response who received at least 2 years of treatment. Switching from an intravenous to an oral bisphosphonate may be a valid option. Patients should be also advised to take calcium and vitamin D. The use of growth factors should be evaluated on single case basis, and long-acting growth factors should be considered.

### Clinical Trials

Involving patients in a clinical trial remains an appropriate strategy to consider for all MM patients, as it allows access to off-label, highly beneficial drugs or combinations that can be otherwise unavailable, especially for patients with advanced disease. Therefore, this participation, once patients have been enrolled, should, in principle, continue. Nonetheless, we recommend every single institution to carefully weigh the advantages and disadvantages of including any single patient in a clinical trial, also considering the spread of SARS-CoV-2 in specific areas of the world.

Nevertheless, patient safety remains the priority. As a consequence, many centers have modified their protocols, allowing, for example, blood draws to be done locally, telemedicine evaluation whenever possible, and home or extended delivery by hospital pharmacies of the medication(s) under investigation, in order to reduce or avoid hospital visits. In general, we do not suggest, despite the current COVID-19 pandemic, to suspend or slow enrollments, if appropriate measures are applied.

Regarding COVID-19 infected MM patients, such a condition should be an opportunity for collecting precious clinical data; performing prospective studies for the management of the infection and its severe complications, should be also stimulated.

### COVID-19 Positive Patients

For COVID-19 positive patients we recommend holding treatment for at least 2 to 3 weeks, depending on the clinical need. Antineoplastic therapy should be reintroduced only after complete convalescence, ensuring safety. Metabolic and bone-related acute complications can be instead managed with adequate supportive care. It is not known whether an IgG positive serology test indicates that myeloma treatment can be initiated as usual.

Currently there are scarce published data regarding both the prevalence and outcomes for patients with MM exposed to COVID-19. In general, we can expect that the COVID-19 infection pattern of MM patients will be similar to that of patients with solid cancers, but no data support this estimation so far. Some data from the International Myeloma Foundation have shown that, until April 30, 2020, few MM patients have tested positive for COVID-19 and are almost all doing well in the Asia-Pacific region, as well in US, with rare exceptions. More COVID-19 cases were observed up to now in Italy, Spain, and France, and some of them died from the infection. Deaths have been reported mostly in fragile elderly patients and in end-stage disease.

A UK survey of 75 COVID-19 positive symptomatic patients with MM receiving active anti-myeloma therapies, whether managed in the inpatients or outpatient setting, has been recently reported (39). At the time of publication, 41 patients (54.6%), had died. Nine patients required critical care support and all deceased. Mortality was associated with multiple comorbidities, older age, and Afro-Caribbean origin. A very few patients received ruxolitinib, tocilizumab, or hydroxychloroquine.

From February 25 to May 18, 2020, 94 hospitalized patients with MM were included in a large, nationwide study of COVID-19 positive patients with hematological malignancies in Italy (NCT04352556). Some of them had admission to intensive care units requiring assisted ventilation or died during the pandemic. Various treatments with antiviral agents, hydroxy-chloroquine, heparin, or tocilizumab were also employed. Detailed data on this study will be soon available.

Clinical experience is accumulating with patients with MM infected by COVID-19. Close monitoring is crucial in SARS-CoV-2 patients in self-isolation at home, with asymptomatic or paucisymptomatic infections, as clinical deterioration may happen at the end of the first week. It is well-known that the risk of thrombosis is already increased in MM patients, especially in the early phase of treatment, at relapse, or in patients receiving thalidomide or lenalidomide treatments. Furthermore, there is increasing evidence that COVID-19 infection *per se* increases the risk of thrombosis and endothelitis. Clinicians should evaluate the risk of thrombosis on single case basis, and evaluate the prophylactic or therapeutic use of anti-thrombotic drugs. Given some positive results with heparin in subjects positive for COVID-19, such a treatment could be a recommended option in these patients.

Until now, no specific clinical trials of COVID-19 directed therapy in MM have been published. Some patients treated with tocilizumab, a monoclonal antibody directed against IL-6 receptor, had a positive outcome (40, 41). In a case we recently observed, however, such a treatment was not effective.

### Conclusions

The COVID-19 era is challenging and the management of MM patients should be based on strict collaboration between patients, clinical staff, health care institutions and families. A summary of the currently available recommendations and a detailed decision-making algorithm for the management of patients with MM during COVID-19 pandemic has been very recently reported by the European Myeloma Network (EMN) (31). Although shared guidelines should be adopted, when possible, many cases will be evaluated on an individual basis, focusing on disease characteristics as well as on patient's history. Any effort should be done to manage MM patients in any phase of the disease to ensure that they will receive the most efficacious treatment, and their prognosis will not be negatively affected by the current pandemic. Ongoing data collection efforts will be fundamental to filling the existing knowledge gaps about the epidemiology, treatment, and outcomes of COVID-19 for MM patients in the near future (42).

## Management of Patients With Lymphoma and CLL

The issues relevant to the management of patients with lymphoma and CLL during the COVID-19 pandemic can be broken down as follows:

1. Diagnosis, staging, risk assessment, and initial laboratory evaluation
2. Treatment decisions in patients who are COVID-negative
  - High grade lymphomas with front line curable options
  - Aggressive curable non-hodgkin's lymphomas and Hodgkin's lymphoma
  - Indolent lymphomas and CLL
3. Treatment decisions in patients who are COVID-positive
4. Supportive care and monitoring of toxicity

### Diagnosis, Staging, Risk Assessment, and Initial Laboratory Evaluation

The diagnosis and initial assessment of patients with suspected or newly diagnosed lymphoma and CLL has to be adapted to the existing isolation and travel restriction rules. In-person visits should be minimized and most visits should be performed via Telehealth, including new patient visits and consultations. Imaging and staging procedures should be consolidated. Biopsy techniques that do not require anesthesia and surgical procedures are preferred, and CT or ultrasound guided core needle biopsies are recommended. FNAs should be avoided. Long term venous access modalities that requires less frequent care should be utilized. The use of chemotherapy-clearance tests in low risk patients who need immediate access to therapy (PFTs/Echocardiogram in young healthy patients) may need to be reconsidered. Infusion centers overcrowding should be avoided. Oral chemotherapy options and home therapy should be pursued.

### Treatment Decisions in Patients Who Are COVID-Negative

#### Aggressive Non-Hodgkin's and Hodgkin's Lymphoma

The cure rate for diffuse large B-cell lymphoma (DLBCL) is ~60–70%, with rituximab-based chemoimmunotherapy, such as R-CHOP or similar combinations (43). These are outpatient regimens given every 3 weeks, do not require interim visits in the absence of complications, and therefore are a good treatment option during the COVID-19 pandemic. Intensive transfusion support is not required in most patients with DLBCL, except in the elderly (44). For patients with double hit lymphomas (DHL) R-CHOP is inadequate and DA-EPOCH-R has emerged as the standard of care in the US. Given the appropriate resources, DA-EPOCH-R can be given as an outpatient in selected patients in the U.S, starting with cycle 2, after inpatient completion of cycle 1 without complications. The same approach is used for patients with primary mediastinal B-cell lymphomas (PMBCL), especially in cases where a radiation-free strategy is desired. At the Sidney Kimmel Cancer Center, since 2018, we have established an active outpatient DA-EPOCH program for DLBCL patients, thanks to a well-organized and robust outpatient home infusion service (Jefferson Home Infusion Service - JHIS). In cases where outpatient DA-EPOCH-R is not doable, R-CHOP-21, with involved field radiation for PMBCL, is an acceptable alternative during the pandemic. Given the availability of subcutaneous formulation, the administration of rituximab is also logistically easier. Despite theoretical concerns that filgrastim may exacerbate the respiratory effects of COVID-19 infection, in cases where severe myelosuppression is expected, growth factor support should be used, with the best option of Peg-filgrastim with on body injector (Onpro^®^).

The management of patients with aggressive lymphoma and high-risk for CNS relapse was a challenge before COVID-19 and the dilemma has now been further exacerbated by the logistical constraints imposed by the pandemic. While intrathecal (IT) chemotherapy with methotrexate, given on the same day of R-CHOP for the first 3–4 cycles, is feasible in the outpatient setting, its efficacy in high risk patient is low, especially in preventing parenchymal involvement, and the recent trend has shifted to providing prophylaxis with high-dose methotrexate (HDMTX), often given on day 15 of each R-CHOP cycle, for 2–4 doses, depending on patients' characteristics and presentation. Since integrating HDMTX with DA-EPOCH-R is extremely challenging, in light of the excessive myelosuppression and non-hematologic toxicity, CNS prophylaxis in patients with DHL who are at very high risk, often has to fall back on IT methotrexate. Alternatively, two doses of HDMTX given after completion of six cycles of DA-EPOCH-R is an option.

Considering that the orchestration of combined modality therapy is logistically more complex than single modality therapy, patients with non-bulky (<10 cm) limited stage DLBCL can be treated with R-CHOP X 4, without the need for radiation or radio-immunotherapy, based on recent data from the FLYER trial (45). In the presence of bulky disease, or when radiation therapy is indicated according to best clinical judgement, hypo-fractionated RT regimens are recommended, according to recent guidelines (46).

For patients with relapsed/refractory DLBCL that is chemosensitive to second-line chemotherapy high-dose chemotherapy (HDC) with ASCT, with minimal or no delays, remains the optimal therapy in light of its curative potential (see section on Cellular Therapy below). There is no preferred second line regimen for DLBCL. Options include oral therapy with lenalidomide-based regimens, chemoimmunotherapy with bendamustine and rituximab, which has the advantage of being administered every 28 days, and R-Gem-Ox which can be administered in split doses every 14 days. Polatuzamab and Bendamustine, approved in the third-line setting in the US) is another option. In light of the logistical complexity and the probability of utilizing scarce ICU beds CART-cell therapy remains minimally used during the pandemic.

Hodgkin's disease is a highly curable malignancy and every effort should be made to avoid treatment delays and maintain dose intensity and keep therapy on schedule. For patients with early stage favorable classic Hodgkin lymphoma (cHL) two cycles of ABVD and limited radiotherapy (20 Gy) remains the standard of care, but–as for limited stage DLBCL–the logistical burden of administering combined modality therapy during the pandemic is high. An alternative is the use of PET-adapted single modality therapy with four cycles of ABVD (47). At the SKCC we are omitting bleomycin from ABVD in early stage patients with a negative interim PET2, extending the data from the RATHL (response-adapted therapy for advanced Hodgkin lymphoma) clinical trial to patients with early stage favorable cHL (48).

For patients with early stage, unfavorable cHL (bulky mediastinal mass, B symptoms, elevated ESR, multiple nodal sites) acceptable standards of care include PET-adapted single modality therapy with ABVD x 4–6 (especially for those who are unfavorable for reasons other than bulky disease) or combined modality therapy with ABVD X 4 + involved site radiotherapy (ISRT). Once again, single modality is preferred, if possible. Omission of bleomycin after a negative PET/CT is also appropriate for these patients.

For patients with advanced stage cHL the recommended approach remains single modality PET-adapted ABVD, with the intent of eliminating bleomycin after cycle 2 in PET2-negative (Deauville 1–3) patients. For patients with a positive (Deauville 4–5) interim PET/CT most centers in the U.S. do not transition immediately to dose-escalated BEACOPP but rather proceed with additional ABVD therapy and repeat a PET/CT. An alternative to this regimen in the US, is AVD with Brentuximab vedotin, according to the recently published ECHELON-1 trial. This regimen has not yet been universally embraced in the US, in part because of the increased myelosuppression, requiring for routine growth factor support, and the high rate of sensory peripheral neuropathy (SPN).

Older patients (variably defined, most often >60 years) with cHL are a high-risk category, with inferior outcomes. AVBD remains the standard of care, with omission of bleomycin in most or all cycles, but with an increased risk of myelosuppression and a high rate of hospitalization. For patients who are frail and elderly, not ASCT eligible, and experience significant toxicity with the first cycle of AVD, single agent brentuximab vedotin (BV) can be explored on a compassionate basis.

During the pandemic, even for fit, young, intent-to-transplant patients with relapsed or refractory cHL, second line regimens that do not require hospitalization such as gemcitabine- and bendamustine-based regimens are preferred. BV or immune checkpoint inhibitors (every 4 weeks) can be used. Our approach at the SKCC is to proceed to ASCT in all patients with R/R cHL who have chemosensitive disease, and our BMT program has remained open. In selected patients with R/R cHL, especially for older patients and patients with late relapses, or at centers where BMT programs have stopped their operations, it is acceptable to consolidate responses to second- or third-line therapy with radiotherapy instead of ASCT.

#### Indolent Lymphomas and CLL

The primary focus for indolent B-cell and T-cell lymphomas (such as CTCL) in the setting of COVID-19 should be safety for the patient and reduction of the risk of exposure for patients and HCT. Consensus guidelines for CLL and indolent lymphomas have long routinely supported watchful waiting and initiation of therapy only when there is a clear indication. These criteria remain valid and should be followed even more strictly during the pandemic. Decisions should be made on a case by case basis and according to best clinical judgement, but if the indication for therapy is questionable, treatment deferral with repeat imaging and close monitoring should be offered to the patient. While the risk benefit calculation of administering single agent rituximab in asymptomatic patients with follicular lymphoma (FL), marginal zone lymphoma (MZL), and other low-grade B-cell lymphomas has often fallen on the side of initiating therapy, especially in patients uncomfortable with treatment deferral, these considerations need to be revised during the pandemic. In light of the possible adverse impact of hypogammaglobulinemia on COVID-19, treatment of asymptomatic patients with rituximab is not recommended and should be discouraged.

When treatment is indicated (49), the risks and benefits of rituximab monotherapy vs. chemo-immunotherapy should be carefully considered. Patients with localized, symptomatic disease should receive single modality radiotherapy. While radioimmunotherapy remains an underutilized treatment option for relapsed and refractory FL, it has the advantage of requiring only a short course of therapy, with high efficacy and should be considered in selected patients. The only available radioimmunotherapy agent at this time is ibritumomab tiuxetan (Zevalin). Patients with indolent B-cell lymphomas and high-risk features according to GELF may have greater clinical benefit from chemo-immunotherapy, such as R-CVP or R-CHOP with growth factor support, rather than single agent rituximab. Maintenance rituximab, with its clinical benefit limited to progression free survival (PFS), is discouraged during the pandemic. If maintenance rituximab is used, immunoglobulin levels should be carefully monitored, especially in older patients. Patients with comorbidities, recent infections, and documented hypogammaglobulinemia, whether or not it is secondary to rituximab, may benefit from monthly IVIG. In COVID-positive patients, especially patients with CLL and recurrent infections, IVIG can be continued, although given the higher risk of thromboembolism during COVID-19, this decision should be made on a case-by-case basis and patients should be monitored for symptoms of thromboembolic disease.

Since initial approvals in 2013, 2014, and 2015 for patients with relapsed Mantle Cell Lymphoma (MCL), CLL/SLL, and Waldenstrom, respectively, ibrutinib has become an essential component of the therapeutic toolbox for these B-cell neoplasms and remains a good treatment option in the US. Ibrutinib appears to be safe during the pandemic and no new signal of toxicity has been reported. In fact, anecdotal cases have suggested that ibrutinib may have a protective effect on COVID-19 pulmonary injury in patients with Waldenstrom (50). Data on more recent BTK inhibitors are not available. One advantage of ibrutinib and other oral agents, such as lenalidomide is that they limit the number of visits to the outpatient clinic, therefore reducing the patient's risk of contracting COVID-19. We have utilized this approach, with labs obtained locally and telehealth visits for most of our patients with indolent lymphomas.

Symptomatic patients with CLL represent a high-risk population during the COVID-19 pandemic because of their underlying immunodeficiency and documented inadequate immune response to infections. These patients are at high risk of bacterial infections and reactivation of latent herpesvirus infections, such as HSV, VZV, CMV, and EBV. Whether or not patients with CLL have a disproportionately higher incidence of severe COVID-19 compared to patients with other malignancies is not known.

As in other indolent B-cell neoplasms, initiation of treatment in CLL patients should be deferred if possible and the iwCLL guidelines should be used to assist with treatment decisions (51) For symptomatic patients who require immediate therapy, treatment decisions should be made based on disease and patient-specific factors, with preference for oral agents and therapies that can be given in the outpatient setting. The goal is to expose the patient to fewer clinic visits and lab assessments. Considering that recent studies in patients with untreated CLL (52, 53) have demonstrated that the addition of rituximab to ibrutinib has thus far not provided added benefit in terms of response rates or survival, avoiding treatment with rituximab, and obinutuzumab in these patients during the pandemic is a very reasonable approach.

The use of another highly effective drug in CLL, venetoclax, has been generally discouraged during the pandemic because it typically requires multiple and extended clinic visits, with dosing ramp up and lab testing, to monitor for tumor lysis syndrome (TLS). However, patients with ibrutinib-resistant CLL and patients with high risk CLL, such as those with 17p deletion, may not have other good treatment options. We have successfully administered venetoclax to elderly patients with refractory CLL in the outpatient setting with remote Telehealth monitoring and frequent blood draws and laboratory monitoring for TLS using Jefferson Home Infusion Services.

While the experience remains anecdotal, it appears that COVID-positive CLL patients who are asymptomatic or have very mild symptoms, can safely continue therapy. However, treatment decisions for patients with more symptomatic COVID-19 have to be based on assessing whether the patient will incur more risk from the CLL or from the theoretical possibility of developing severe COVID complications, due to therapy. Although there is a degree of consensus that anti-CD20 monoclonal antibodies should be held in COVID-positive CLL patients, because of the concern for severe hypogammaglobulinemia, treatment decisions, such as holding or continuing therapy, should be made on a case-by-case basis. For patients receiving BTK inhibitors, discontinuation can result in CLL flares and cytokine release that mimic COVID-19, so judgment should be exercised and COVID-19 testing may need to be included in the treatment algorithm.

## Management of Patients Receiving Stem Cell Transplants and Cellular Therapy

The COVID-19 pandemic posed unique challenges to hematopoietic cell therapy (HCT) and immune effector cell therapy (IECT) programs, not only in the United States but on an unprecedented global level. The inherent medical complexity of these procedures, coupled with the intricate network of logistical, operational, and regulatory steps they require, had to be approached with agile and frugal planning. *Agility*, because of all the obfuscations surrounding the pandemic timeline, the initial lack of availability of COVID-19 testing, and later on antibody testing, and an interminable list of uncertainties and moving sand. *Frugality* because of the strain that the pandemic caused on resources, staff, and on the stem cells and effector T cells supply chain. COVID-19 specific guidelines for HCT from the American Society of Transplantation and Cellular Therapy (ASTCT) and the European Bone Marrow Transplant (EBMT) are rapidly evolving. However, these recommendations do not account for transplant centers' variability in terms of available resources or surge patterns. Laid bare, this pandemic took everyone by surprise! On March 13, 2 days after the WHO declared the COVID-19 a pandemic, we devised a congruent model to deal with the new reality. For this brief introduction, we will summarize The Sidney Kimmel Cancer Center (SKCC) program's experience (for context), with a focus on three particular aspects:

1. Managing the workflow
2. Deferring IECT and non-urgent high dose chemotherapy (HDC) procedures
3. Securing safe allogeneic stem cell grafts

### The Workflow of the Clinical Care Team

We designed a two-team model, one for managing the inpatient unit and the second to carry out the outpatient operation. Continuity of care was maintained through virtual communication between the two teams. Additionally, members of the same team limited their in-person interactions to the absolute minimum. Approximately 75% of in-person clinic visits were effectively converted to our institution's telehealth platform, a silver lining of this pandemic that will likely stay with us. We limited the exposure of hospitalized patients to a minimal number of the inpatient care team, in the vast majority of cases, the daily physical interaction was limited to one nurse and one physician excluding consulting services when needed. Daily roundtable morning rounds were leaned out to the bare minimum of clinical staff with virtual participation from ancillary services. All incoming new or re-admitted patients were properly screened for COVID-19 before entering the inpatient unit, and a strict no visitors policy was implemented. Finally, we retained the nursing staff to work exclusively in the inpatient unit and eliminated the need for nurse floaters from other services. This comprehensive model effectively limited the risk of COVID-19 nosocomial exposure and ensured adequate backup coverage for team members who may get infected or need to be quarantined for inadvertent exposure. As of the timing of writing this manuscript, on June 1, 2020, our inpatient unit remains COVID-19 negative, and none of our clinical staff members or patients experienced nosocomial exposure.

### IECT Procedures and HDC Procedures

Our IECT portfolio includes industry-sponsored actively accruing chimeric antigen receptor T-cell (CAR T) trials for CLL, MM, metastatic castration-resistant prostate cancer, and a variety of solid tumors. We are also an approved center for the two commercial CAR T products. Since mid-March 2020, all industry-sponsored research and commercial CAR T procedures were suspended at our institution. Patients who were eligible for this treatment were treated with alternative therapeutic modalities or kept under close monitoring. Screening for CAR T research protocols continued in order to identify patients who should be prioritized in the post-surge phase.

All eligible lymphoma patients proceed to HDC with no deferment. All eligible multiple myeloma and amyloid disease patients for HDC were deferred for a brief period. However, on April 21, 2020, we decided to proceed with treating all the deferred cases. Many factors contributed to the reversal of the deferment and resumption of HDC procedures. Our institution's projection for COVID-19 infection in the Philadelphia area indicated a flatter more prolonged surge curve compared to a steeper curve in neighboring New York City. The improved and wide availability of the rapid COVID-19 testing allowed for more efficient screening of all patients on the admission day. Finally, our piloted workflow management strategy has proven to be safe and effective in creating enough capacity to handle all deferments.

### Securing Safe Allogeneic Stem Cell Grafts

No routine COVID-19 testing was provided for asymptomatic HCT donors. Once patients were started on their conditioning regimen, donors were instructed to self-quarantine and report any suspicious symptoms. All matched and unmatched related donor HCT procedures proceeded without deferment per our program post-transplant cyclophosphamide (PTCy) T cell tolerization two-step protocols (54).

Consistent with the recommendations of the national marrow donor program (NMDP), cryopreservation was required for all unrelated donor stem cell grafts. In line with the recent Center for International Blood and Marrow Transplant Research (CIBMTR) data (55) we successfully utilized post-transplant high dose cyclophosphamide after cryopreserved stem cell products with proper, timely engraftment and no increased short-term complications.

## Conclusions

The COVID-19 pandemic presents a challenge that is unparalleled in recent times and has created unprecedented medical, logistical, financial, and public health hurdles to the delivery of optimal care for patients with cancer, and in particular those with hematologic malignancies. While robust data are still missing, preliminary observations, and experience with other endemic or seasonal viral respiratory infections suggest that the greater degree of immunocompromise, due to either the primary malignancy or the severe myelosuppression and lymphodepletion associated with intensive chemotherapy regimens, is likely to expose patients with hematologic malignancies to a greater risk of SARS-CoV-2 infection and severe clinical outcomes during COVID-19. Curative therapy for many patients with hematologic malignancies requires the use of dose intensive chemo-radiation protocols, followed by autologous or allogeneic SCT. Health care systems have rapidly adapted to these challenges and access to life saving and life prolonging therapies has remained available. The technology explosion of Telehealth and home care platforms has allowed patients to remain in contact with their HCT and has allowed management and follow up of patients requiring therapy during the pandemic. While routine elective procedures were deferred for a while, scheduling and performance of the diagnostic, staging, and chemotherapy clearance procedures has resumed, but much was learned in terms of same day consolidation all the diagnostic and staging procedures. In some cases, what was once thought to be impossible has become a normal routine. COVID-testing platforms have been expanded remarkably, and serological testing is becoming more available. Clinical trial operations have remained open, if scaled down, in many institutions during the pandemic, and study logistics, including consenting, sample acquisition and transportation, and study visits have been made more flexible. While the peak of the pandemic may soon be past, in the absence of a vaccine, the risk of additional waves of infection remain, and our level or alertness and the operational response to future challenges need to be maintained.

## Author Contributions

All authors listed have made a substantial, direct and intellectual contribution to the work, and approved it for publication.

## Conflict of Interest

The authors declare that the research was conducted in the absence of any commercial or financial relationships that could be construed as a potential conflict of interest.
